# Cardiovascular complications as a consequence of coronavirus infection in pregnant women managed in ambulatory settings

**DOI:** 10.3389/fcvm.2026.1907396

**Published:** 2026-10-15

**Authors:** Ilia Davarashvili, Ariel Israel, Shlomo Vinker, Hila Hochler

**Affiliations:** 1 Cardiology Department, Leumit Health Care, Jerusalem, Israel; 2 O2 Health Promotion and Sports Medicine Department, The Howard and Mary Edith Cosell Association for Physical Education, The Safra Sports Center, Hebrew University, Jerusalem, Israel; 3 Department of Epidemiology and Preventive Medicine, Gray Faculty of Medical & Health Sciences, Tel Aviv University, Tel-Aviv, Israel; 4 Israel and the Department of Family Medicine Gray Faculty of Medical & Health Sciences, Leumit Health Care, Tel Aviv University, Tel Aviv, Israel; 5 Adelson School of Medicine, Ariel University, Ariel, Israel; 6 Department of Obstetrics and Gynecology, Laniado Medical Center, Netanya, Israel

**Keywords:** ambulatory settings, cardiovascular complications adverse events, COVID-19, deep vein thrombosis, last menstrual period, myocarditis, pregnant women, pulmonary embolism

## Abstract

**Background:**

SARS-CoV-2 has been linked to cardiovascular complications in the general population. However, data regarding cardiovascular outcomes among pregnant women remain limited, particularly in community-based ambulatory populations, as most previous studies focused primarily on hospitalized or critically ill patients. In this retrospective population-based matched cohort study, we evaluated whether COVID-19 infection during pregnancy is associated with an increased incidence of cardiovascular complications among pregnant women managed predominantly in outpatient settings.

**Methods:**

We conducted a retrospective population-based matched cohort study using electronic health record data from pregnant women between 2020 and 2025. Women with COVID-19 during pregnancy were matched 1:1 to women without documented COVID-19 during pregnancy according to demographic, obstetric, and clinical characteristics. The final analytical cohort included 4,068 women in each group. Cardiovascular outcomes during pregnancy and within a year since last menstrual period were assessed. Baseline demographic and obstetric characteristics were comparable between the COVID-19 and control groups. No significant differences were observed between the groups in the incidence of major cardiovascular complications, either when analyzed individually or as a composite endpoint (0.15% vs. 0.15%; OR 1.00, 95% CI 0.27–3.74). Specifically, there were no significant differences in the occurrence of atrial fibrillation/flutter, congestive heart failure, cardiomyopathy, myocarditis, pulmonary embolism, or deep venous thrombosis. In contrast, cough and backache were significantly more frequent among women with COVID-19 than among matched controls (6.88% vs. 4.87%; OR 1.45, 95% CI 1.19–1.75; 13.8% vs. 11.8%; OR 1.20, 95% CI 1.05–1.37; *p* = 0.007 respectively).

**Conclusions:**

Our findings suggest that among pregnant women in ambulatory community settings, COVID-19 infection is not associated with an increased risk of major cardiovascular complications.

## Introduction

The novel coronavirus disease (COVID-19) was declared a global public health emergency by the World Health Organization on March 11, 2020 (1–3). As of the COVID-19 updated registry, 422 million confirmed cases have been reported globally to for February 2022, with 5 million deaths worldwide.

The main clinical manifestation of COVID-19 disease is characterized by acute respiratory distress syndrome and atypical pneumonia (4–7). Emerging studies have demonstrated the harmful effects of SARS-CoV-2 on the cardiovascular (CV) system, such as myocardial injury, which is associated with myocardial inflammation and damage (8, 9). It has been suggested that heart injury caused by COVID-19 infection might be an important cause of severe clinical phenotypes or adverse events in affected patients (10–12). Myocardial damage is closely related to the severity of the disease and subsequent prognosis in patients with COVID-19 (10, 13–15). In nonpregnant women, studies have shown that severe acute respiratory syndrome due to coronavirus causes cardiac injury, which can result in myocardial inflammation and damage (4). Almost 33% of nonpregnant patients with COVID-19 admitted to the intensive care unit (ICU) developed cardiac injury (14). In addition to direct myocardial injury and hypoxia-related damage, accumulating evidence indicates that cardiovascular complications of COVID-19 are mediated by COVID-19-associated coagulopathy (CAC), a complex syndrome characterized by endothelial dysfunction, platelet activation, immunothrombosis, and dysregulated coagulation pathways (16–18). Rather than being solely a consequence of severe respiratory disease, myocardial injury may result from diffuse microvascular thrombosis, endothelial inflammation, and thromboembolic phenomena involving both the arterial and venous circulation. Pregnancy itself represents a physiological hypercoagulable state; therefore, the interaction between pregnancy-associated coagulation changes and CAC provides an additional biologically plausible mechanism for cardiovascular complications during SARS-CoV-2 infection (17–19).

During pregnancy, several changes occur in maternal physiology: raise in blood volume, cardiac output, baseline heart rate and high end diastolic pressure (20–22). This population is also more prone to tachyarrthmias. All these physiological shifts may lead to suboptimal cardiac adaptation and overt clinical heart failure (20–22). Moreover, cardiovascular disease (CVD) is the leading cause of death during pregnancy, accounting for approximately 33% of the maternal deaths in the United States (23).

Despite numerous studies investigating the effect of COVID-19 on the adult heart, little is known regarding its impact on pregnant women (4). Early reports suggested that pregnant women represented a vulnerable population requiring careful surveillance (24). Although severe cardiovascular complications have been described primarily among hospitalized patients with moderate-to-severe COVID-19, relatively little is known regarding cardiovascular outcomes among pregnant women managed predominantly in community ambulatory settings (17, 18, 25).

The objective of this study is to investigate the incidence, predictors, and outcomes of cardiovascular complications in a pregnant population with COVID-19 infection within a community setting.

## Methods

### Study design and data source

We conducted a retrospective, population-based, matched cohort study using longitudinal electronic health records (EHRs) from Leumit Health Services (LHS), a nationwide health maintenance organization in Israel that serves approximately 730,000 members. The study population included female LHS members aged 16–45 years with a documented pregnancy and available EHR data between January 1, 2020, and December 31, 2025.

### Pregnancy cohort definition

Pregnancies were identified using the LHS pregnancy timeline algorithm, which integrates pregnancy-related records, estimated last menstrual period (LMP), trimester dates, pregnancy end date, number of fetuses, and newborn information. Only pregnancies with a documented second-trimester date and a pregnancy end date within the member's observable EHR period were eligible. Follow-up eligibility was restricted according to LHS membership, death, cancellation date, and the predefined reproductive-age window.

### Exposure definition

The exposed group included women whose first recorded COVID-19 diagnosis occurred during pregnancy. COVID-19 during pregnancy was defined by an overlap between the first documented COVID-19 episode and the pregnancy interval, allowing for infection occurring from 30 days after the estimated LMP through pregnancy end. COVID-19 infection status was identified using a centralized national COVID-19 registry established by the Israeli Ministry of Health, which integrated results from clinic-based and self-administered PCR tests performed during the study period. Women without a documented COVID-19 diagnosis during pregnancy formed the pool of potential controls.

### Covariates and matching variables

Baseline covariates were derived from EHR data before or during early pregnancy and included age at pregnancy, birth year, year of first available EHR record, socioeconomic status (SES), ethnic sector, BMI category, LMP quarter, number of fetuses, primigravida status, and the number of COVID-19 vaccine doses received before pregnancy. BMI was obtained from recorded weight and height measurements before or near the onset of pregnancy; the BMI category was assigned using the closest available value before the second-trimester date, with fallback to the nearest subsequent value when needed.

### Matching

Cases were matched 1:1 without replacement to controls without documented COVID-19 during pregnancy using an iterative exact-matching procedure. Matching variables included ethnic sector, socioeconomic status (SES), year of first available EHR record, birth year, BMI category, number of fetuses, number of prior COVID-19 vaccine doses, LMP quarter, and primigravida status. Within eligible matching strata, candidate controls were ranked according to proximity in maternal age at pregnancy.

To increase the number of successfully matched cases while maintaining close covariate balance, unmatched cases underwent successive matching iterations in which the grouping boundaries and granularity used for birth year and year of first EHR record were varied, and SES categories were progressively regrouped when exact matches were unavailable. The remaining matching variables were retained throughout the matching procedure. Controls were selected without replacement, such that each control could be included only once. The final matched cohort comprised 4,068 COVID-19-exposed women and 4,068 matched controls.

Socioeconomic status was derived from the residential area socioeconomic index recorded in the LHS database. Ethnic sector was categorized according to LHS demographic classifications. The number of prior COVID-19 vaccine doses was calculated according to the number of vaccine doses administered at least 7 days before the first-trimester date. Primigravida status was defined as the first recorded pregnancy. The LMP quarter was derived from the estimated last menstrual period date and used to account for temporal and seasonal differences during the pandemic period. The number of fetuses was determined using pregnancy and delivery records.

### Outcomes

The primary outcome was a composite of major adverse cardiovascular events, defined as myocarditis, cardiomyopathy, atrial fibrillation or flutter, congestive heart failure, myocardial infarction, pulmonary embolism, or deep venous thrombosis. Secondary outcomes included each individual component of the primary composite endpoint, as well as selected non-major clinical symptoms and diagnoses. An outcome was considered present if at least one clinical encounter coded for that condition was recorded between 30 days after the estimated LMP and 365 days thereafter; records predating this window did not contribute. Because exposure was classified at the pregnancy level, the outcome window was anchored to the estimated LMP rather than to the date of SARS-CoV-2 infection. Accordingly, outcomes recorded within this window were not necessarily subsequent to the documented infection date.

Outcomes were additionally stratified by first trimester (LMP + 30 d to 13w6d), second trimester (14w0d–27w6d), third trimester (28w0d to delivery), early postpartum (delivery to +42 d) and late postpartum (+43 d to one year from LMP), with person-time computed per interval.

### Statistical analysis

Baseline characteristics and outcomes were compared between the matched COVID-19-exposed and unexposed groups. Continuous variables were summarized as mean and standard deviation (SD) and compared using Student's t-tests. Categorical variables were summarized as counts and percentages and compared using the Fisher's exact test or chi-squared tests as appropriate. Statistical significance was defined as a two-sided *p*-value < 0.05. Statistical analyses were performed using R software version 4.3 (The R Foundation for Statistical Computing). Exact 95% confidence intervals for odds ratios were computed by the conditional maximum likelihood method. Incidence rates per 1,000 person-years are reported with exact Poisson confidence intervals.

Prespecified sensitivity analyses restricted the cohort to unvaccinated pregnancies and stratified it by pandemic period, using an estimated LMP before 2022 to define the pre-Omicron period and 2022 onward for the Omicron-dominant period. To assess the robustness of the findings to the matched study design, we additionally performed sensitivity analyses explicitly accounting for the 1:1 matched-pair structure, using conditional logistic regression stratified by matched-pair identifier and exact McNemar tests based on discordant matched pairs.

## Results

Between 2020 and 2025, 5,879 candidate pregnancies with a first documented COVID-19 infection during pregnancy were identified, of which 5,776 met the final EHR eligibility criteria. A separate pool of 48,965 eligible pregnancies without documented COVID-19 during pregnancy was identified as potential controls, resulting in 54,741 women available for matching. Of the 5,776 eligible COVID-19-exposed women, 4,068 were successfully matched 1:1–4,068 controls, yielding a final analytical cohort of 8,136 women (Figure 1).

**Figure 1:**
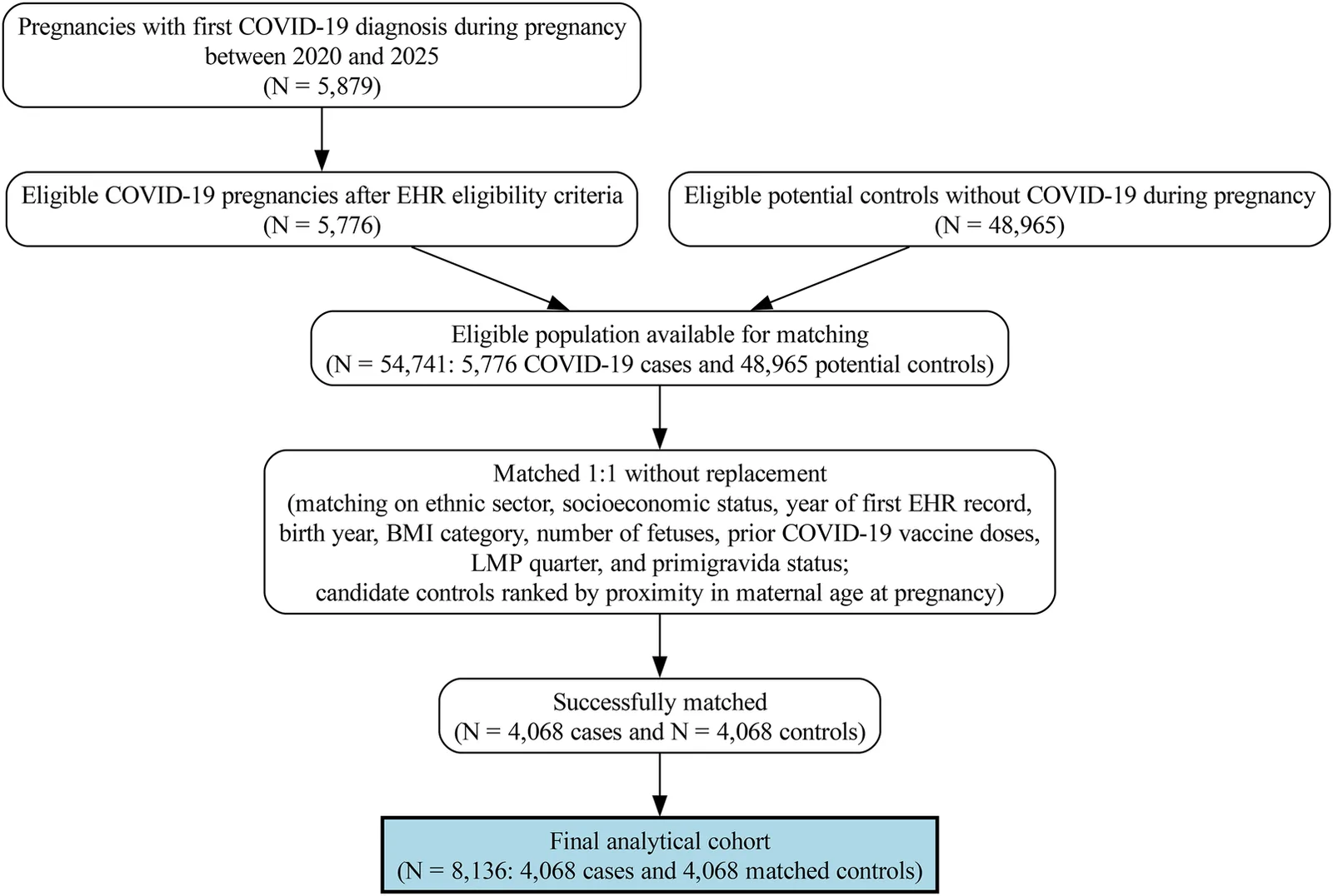
Study population selection and propensity-matched cohort construction. The study cohort was derived from pregnancies with a first diagnosis of COVID-19 during pregnancy between 2020 and 2025. Following application of predefined EHR eligibility criteria, 5,776 COVID-19 pregnancies were identified and considered for matching with 48,965 eligible potential controls without COVID-19 during pregnancy. A 1:1 matching procedure without replacement was performed based on key maternal, sociodemographic, and pregnancy-related characteristics, with candidate controls prioritized according to proximity in maternal age. This process yielded a final analytical cohort of 8,136 pregnancies, comprising 4,068 COVID-19 cases and 4,068 matched controls.

The matching process effectively balanced the two groups, with no significant differences in baseline demographic or clinical parameters (Table 1). The mean age was 28.79 ± 5.39 years in the study group compared to 28.67 ± 5.38 years in the control group (*p* = 0.304). Pre-pregnancy weight was also similar (68.28 ± 14.44 kg vs. 68.63 ± 14.69 kg; *p* = 0.284). Chronic comorbidities were well-distributed between groups, including pre-existing hypertension (9.05% vs. 8.81%; *p* = 0.726), smoking (6.95% vs. 7.64%; *p* = 0.249), and diabetes mellitus (0.93% vs. 0.69%; *p* = 0.266). Pre-existing cardiac conditions, such as a history of valve disease (0.39% vs. 0.49%) and prior myocarditis (0.02% vs. 0.05%), showed no statistical variation at baseline.

**Table 1:** Baseline characteristics and cardiovascular and other clinical outcomes during 1-year follow-up after the last menstrual period in the study and control groups.

Patient characteristics before pregnancy	Study group = 4068	Control group = 4068	Odds Ratio (95% CI)	*p* value
Age (years, mean ± SD)	28.79 ± 5.39	28.67 ± 5.38		0.304
Weight (kg)	68.28 ± 14.44	68.63 ± 14.69	1.00 [0.13–7.47]	0.284
Hypertension	368 (9.05%)	358 (8.81%)	1.03 [0.88–1.20]	0.726
Smokers	282 (6.95%)	310 (7.64%)	0.90 [0.76–1.07]	0.249
Diabetes mellitus (%)	38 (0.93%)	28 (0.69%)	1.36 [0.81–2.31]	0.266
Renal dysfunction (%)	6 (0.15%)	4 (0.10%)	1.50 [0.36–7.24]	0.754
Obstructive sleep apnea (%)	8 (0.20%)	11 (0.27%)	0.73 [0.25–1.99]	0.647
History of valve disease (%)	16 (0.39%)	20 (0.49%)	0.80 [0.39–1.63]	0.617
History of stroke (%)	1 (0.02%)	1 (0.02%)	1.00 [0.01–78.47]	1.000
Prior myocardial infarction (%)	0 (0.00%)	1 (0.02%)	0.00 [0.00–39.00]	1.000
Myocarditis (%)	1 (0.02%)	2 (0.05%)	0.50 [0.01–9.61]	1.000
Pulmonary embolism(%)	2 (0.05%)	1 (0.02%)	2.00 [0.10–117.97]	1.000
Hypothyroidism(%)	162 (3.98%)	172 (4.23%)	0.94 [0.75–1.18]	0.615
Connective tissue disease (%)	47 (1.16%)	36 (0.88%)	1.31 [0.83–2.08]	0.270
IBD (%)	36 (0.88%)	31 (0.76%)	1.16 [0.70–1.95]	0.624
Multiple sclerosis (%)	3 (0.07%)	6 (0.15%)	0.50 [0.08–2.34]	0.508
ITP (%)	14 (0.34%)	12 (0.29%)	1.17 [0.50–2.77]	0.845
Complications, 1 year follow-up after LMP				
Atrial fibrillation (%)	0 (0.00%)	0 (0.00%)	-	-
Atrial flutter (%)	0 (0.00%)	0 (0.00%)	-	-
Cardiomyopathy (%)	1 (0.02%)	1 (0.02%)	1.00 [0.01–78.5]	1.000
Congestive heart failure (%)	0 (0.00%)	1 (0.02%)	0.00 [0.00–39.00]	1.000
Myocarditis (%)	0 (0.02%)	3 (0.07%)	0.00 [0.00–39.00]	0.625
CVA (%)	1 (0.02%)	1 (0.02%)	1.00 [0.01–78.47]	1.000
Any valvular disease (%)	17 (0.42%)	21 (0.52%)	1.00 [0.01–78.47]	0.626
Pulmonary embolism(%)	1 (0.07%)	0 (0.00%)	Inf [0.03 - Inf]	1.000
Chest pain (%)	86 (2.11%)	65 (1.60%)	1.33 [0.95–1.87]	0.100
Deep venous thrombosis (%)	4 (0.10%)	1 (0.02%)	4.00 [0.40–197]	0.375
Hypertension (%)	50 (1.23%)	40 (0.98%)	2.40 [0.79–8.72]	0.340
Worsening of renal function (%)	7 (0.17%)	4 (0.10%)	Inf [0.03 - Inf]	0.549
Diabetes mellitus (%)	57 (1.40%)	40 (0.98%)	1.59 [0.73–3.59]	0.102
Hypothyroidism (%)	192 (4.72%)	215 (5.29%)	0.70 [0.42–1.14]	0.263
ITP (%)	22 (0.54%)	16 (0.39%)	2.00 [0.54–9.09]	0.417
New diagnosis of IBD (%)	2 (0.05%)	3 (0.07%)	0.67 [0.06–5.82]	1.000
Backache(%)	562 (13.8%)	480 (11.8%)	1.20 [1.05–1.37]	0.007
Cough (%)	280 (6.88%)	198 (4.87%)	1.45 [1.19–1.75]	<0.001
Headache (%)	249 (6.12%)	210 (5.16%)	1.20 [0.99–1.45]	0.068
Composite of cardiovascular complications	6 (0.15%)	6 (0.15%)	1.00 [0.27–3.74]	1.000

### Cardiovascular and systemic complications (1-year follow-up)

Major cardiovascular events were rare and equally distributed: six events occurred in each group over the one-year window (0.15% vs. 0.15%; OR 1.00, 95% CI 0.27–3.74). Myocarditis was not recorded in COVID-19 patients and two cases in the control group; cardiomyopathy in one patient per group; congestive heart failure in one control; pulmonary embolism in one COVID-19 patient; and deep venous thrombosis in four COVID-19 patients and one control (OR 4.00, 95% CI 0.40–197). No atrial fibrillation or flutter was recorded in either group during follow-up. Chest pain did not differ significantly (2.11% vs. 1.60%; OR 1.33, 95% CI 0.95–1.87; *p* = 0.10). Stratification by trimester and postpartum period showed no consistent temporal pattern for any cardiovascular outcome (Supplementary Table 1, Figure 2). These findings were unchanged in analyses restricted to unvaccinated pregnancies and repeated within each pandemic period (Supplementary Table 3).

**Figure 2:**
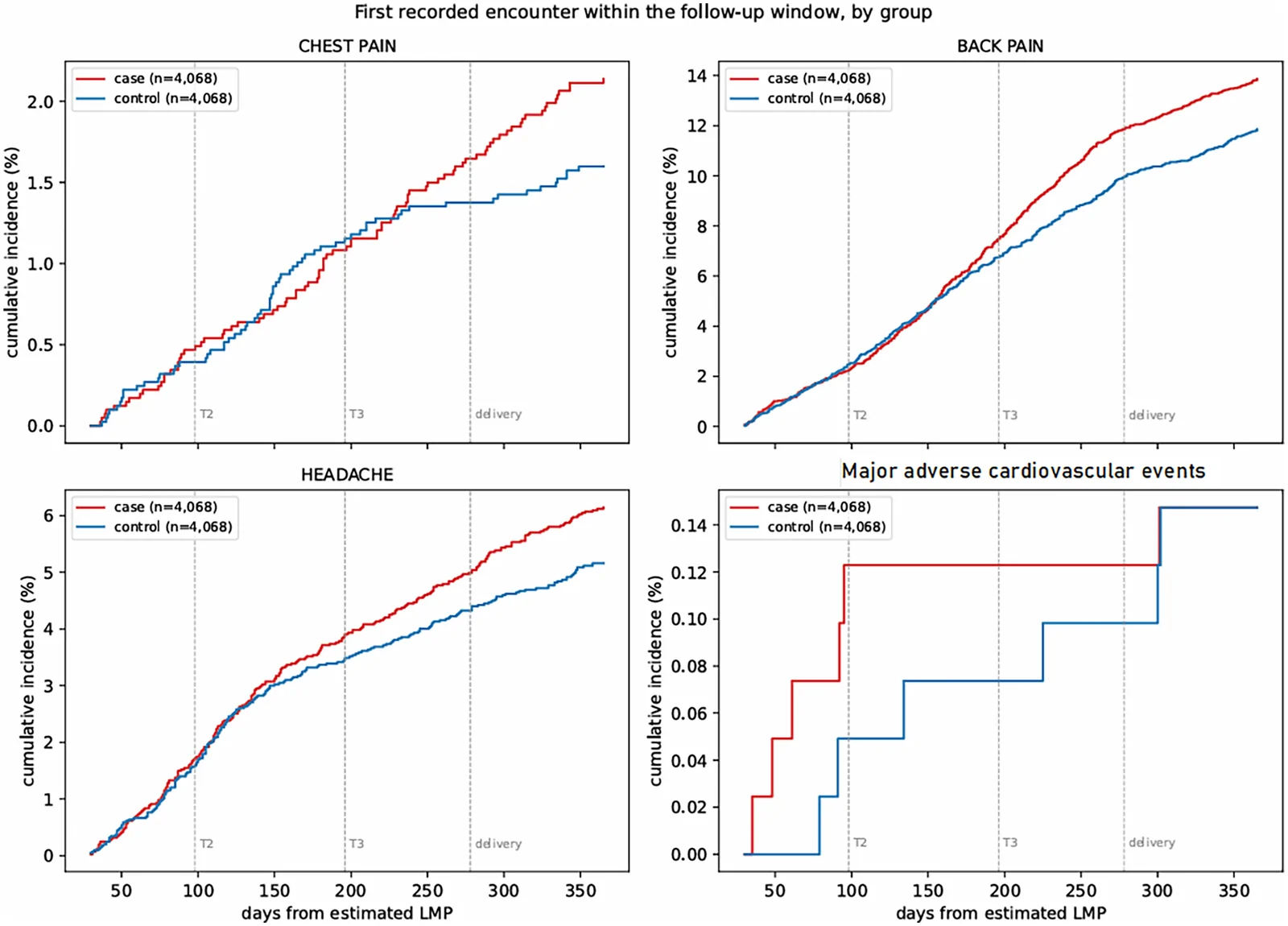
Cumulative incidence of first recorded chest pain, back pain, headache, and major adverse cardiovascular events during pregnancy among individuals with COVID-19 (case group; n = 4,068) and matched controls (n = 4,068). Curves show cumulative incidence according to days from the estimated last menstrual period (LMP). Vertical reference lines indicate pregnancy trimesters and delivery. MACE, major adverse cardiovascular events.

Recorded cough was more frequent in the COVID-19 group (6.88% vs. 4.87%; OR 1.45, 95% CI 1.19–1.75; *p* < 0.001), with balanced baseline prevalence (37.6% vs. 38.5%; *p* = 0.45), indicating that symptom ascertainment was sensitive to differences in recorded morbidity between the groups. Backache was recorded in 13.8% of the COVID-19 group versus 11.8% of controls (OR 1.20, 95% CI 1.05–1.37; *p* = 0.007); baseline prevalence was, however, already higher in the COVID-19 group (49.5% vs. 46.1%; *p* = 0.003). Headache did not differ significantly (6.12% vs. 5.16%; OR 1.20, 95% CI 0.99–1.45; *p* = 0.068).

The incidence of other systemic conditions remained comparable between the two cohorts; new diagnoses of hypertension (1.23% vs. 0.98%; *p* = 0.340), diabetes mellitus (1.40% vs. 0.98%; *p* = 0.102), and worsening of renal function (0.17% vs. 0.10%; *p* = 0.549) did not show statistically significant differences. Autoimmune and hematological findings, such as ITP and a new diagnosis of IBD, were also similarly distributed (Table 1).

Sensitivity analyses explicitly accounting for the 1:1 matched-pair structure yielded results consistent with the primary analyses. For the composite major cardiovascular outcome, six discordant pairs had an event only in the COVID-19-exposed woman and six had an event only in the matched control, corresponding to a conditional OR of 1.00 (95% CI 0.32–3.10; *p* = 1.00; exact McNemar *p* = 1.00). The matched analyses similarly did not materially alter the findings for chest pain, cough, back pain, or headache.

## Discussion

The primary objective of this study was to evaluate the impact of COVID-19 infection during pregnancy on cardiovascular outcomes in ambulatory settings. In this large-scale, retrospective population-based matched cohort study of 8,136 pregnant women, we found that COVID-19 infection during pregnancy is associated with a modest excess of recorded respiratory and musculoskeletal symptoms, but does not appear to increase the risk of serious objective cardiovascular complications in the general ambulatory population, such as myocarditis, acute coronary syndrome or any type of cardiomyopathy (26).

Pregnancy is characterized by profound hemodynamic adaptations, including a 40%–50% increase in blood volume, augmented cardiac output, and an elevated baseline heart rate (20–22). Collectively, these changes impose a substantial physiological “stress test” on the maternal cardiovascular system. It was therefore hypothesized that the addition of an acute viral infection such as SARS-CoV-2—known to cause respiratory compromise, hypoxia, and potential direct myocardial injury—might overwhelm this finely balanced state. Specifically, we postulated that this “double hit,” comprising the hemodynamic burden of pregnancy alongside the inflammatory and hypoxic effects of COVID-19, would lead to an increased incidence of heart failure and arrhythmias. Contrary to this expectation, our findings suggest that the maternal cardiovascular reserve demonstrates considerable resilience, effectively compensating for these combined stressors in the majority of cases.

Although several studies have examined the cardiovascular consequences of SARS-CoV-2 infection during pregnancy, most have focused on women with severe disease, hospitalization, intensive care, or the peripartum period, populations exposed to greater physiological and cardiovascular stress than the broader ambulatory pregnant population (27–29). Data regarding cardiovascular outcomes among ambulatory pregnant women remain more limited. In this context, our findings are consistent with those of Bikdeli et al., who specifically evaluated pregnant women with COVID-19 managed in the outpatient setting in the multicenter CORONA-VTE-Network study. Among 169 pregnant outpatients followed for 90 days, two venous thromboembolic events occurred and no non-thrombotic cardiovascular events were observed (30). These findings provide relevant context for our observation that clinically significant cardiovascular events were uncommon among pregnant women with predominantly ambulatory COVID-19.

Metz et al. evaluated the association between SARS-CoV-2 infection and a composite outcome of serious maternal morbidity and mortality from obstetric complications in pregnant and postpartum individuals at 17 US hospitals (27). The composite outcome included a broad range of severe obstetric and systemic complications, including hemorrhage, hypertensive complications, sepsis, respiratory complications, thromboembolism, myocardial infarction, peripartum cardiomyopathy, and intensive care admission. Their findings demonstrated an increased risk of serious maternal morbidity and mortality associated with SARS-CoV-2 infection, particularly in the setting of more clinically significant disease. However, this study was not designed to establish specific increase in cardiovascular complications (27). Ferrara et al. investigated a large community-based population of pregnant individuals in California and reported an increased risk of severe maternal morbidity associated with SARS-CoV-2 infection (29). However, severe maternal morbidity represents a composite outcome encompassing numerous obstetric, respiratory, thrombotic, and systemic complications rather than a specific cardiovascular endpoint. Their findings therefore provide important evidence regarding overall maternal morbidity but are not directly comparable with our analysis of specific cardiovascular diagnoses. Furthermore, their follow-up was centered on pregnancy and delivery outcomes, whereas our study provides longitudinal follow-up across pregnancy and into the postpartum period, allowing assessment of potential cardiovascular manifestations beyond the delivery hospitalization (29).

The differences between these studies and our findings likely reflect differences in outcomes, study populations, timing of assessment, and disease severity. Most published studies reporting increased cardiovascular complications in pregnant women with moderate-to-severe COVID-19, whereas our cohort predominantly comprised community-dwelling pregnant women managed in outpatient settings. It is essential to note that previous analysis focused specifically on peripartum admissions—a period characterized by peak hemodynamic stress. Consequently, our findings extend the existing literature by suggesting that cardiovascular risk observed in hospitalized cohorts should not necessarily be extrapolated to ambulatory pregnant populations with generally mild disease (31–36). Furthermore, large population-based studies have demonstrated that increased long-term cardiovascular risk following SARS-CoV-2 infection is also present among non-hospitalized adults, although the absolute risk remains considerably lower than that observed following severe infection. Whether pregnancy modifies this relationship remains incompletely understood (17, 27–29).

Our study provides a crucial perspective by examining a broad, community-based cohort of pregnant women. Unlike studies based on hospital registries or high-acuity cases, our findings indicate that for the vast majority of pregnant women in an ambulatory setting, the clinical course of COVID-19 regarding cardiovascular complications is generally favorable. The principal findings of this study were an increased incidence of cough and backache, accompanied by a trend toward increased rates of chest pain and headache among women with COVID-19. These symptom differences may be considered in the context of post-acute manifestations described following COVID-19; however, the present study did not apply a formal Long-COVID definition and cannot establish that these symptoms represent Long COVID. Recent studies describing post-acute sequelae of COVID-19 have reported persistent chest pain, palpitations, dyspnea, and exercise intolerance in the absence of clinically apparent structural heart disease. Proposed mechanisms include persistent endothelial dysfunction, coronary microvascular impairment, autonomic dysregulation, low-grade inflammation, and residual immune activation. These mechanisms may explain why women in our cohort reported musculoskeletal and respiratory symptoms more frequently (37).

At the same time, the absence of a statistically significant increase in pulmonary embolism or deep vein thrombosis in our cohort should not be interpreted as evidence that SARS-CoV-2 infection does not increase thrombotic risk. Several large population-based studies have reported an increased relative risk of venous thromboembolism (VTE) during pregnancy and the postpartum period following SARS-CoV-2 infection, particularly during the weeks immediately after infection. Ferrara et al., in a large California population-based cohort, reported an association between SARS-CoV-2 infection and severe maternal morbidity, with thromboembolic complications contributing to the observed excess morbidity (29). Bruno et al. subsequently reported an adjusted hazard ratio of 2.62 (95% CI, 1.60–4.29) for VTE from pregnancy through six weeks postpartum among women with SARS-CoV-2 infection (38). Similarly, Örtqvist et al., using population-based Swedish and Norwegian registry data, reported an increased incidence of VTE following COVID-19 during pregnancy, with the greatest relative risk occurring within the first two weeks after infection (adjusted hazard ratio, 4.63; 95% CI, 2.71–7.90), although the absolute number of events remained low (39). Thus, increased relative risk and low absolute event rates are not mutually exclusive. In our predominantly ambulatory cohort, the relatively low absolute incidence of pulmonary embolism and deep vein thrombosis may have limited the detection of differences in these uncommon outcomes. Accordingly, our findings should be interpreted as demonstrating that clinically apparent cardiovascular and thromboembolic events were uncommon in this predominantly ambulatory population, rather than as evidence that SARS-CoV-2 infection has no effect on thrombotic risk during pregnancy.

Taken together, the available literature suggests a nuanced relationship between COVID-19 and cardiovascular health during pregnancy. In ambulatory pregnant women, serious cardiovascular events such as myocarditis, acute coronary syndrome, and cardiomyopathy appear to be uncommon. At the same time, epidemiological studies indicate that SARS-CoV-2 infection may increase the relative risk of VTE, particularly during the early period following infection, even when the absolute number of events remains low (30, 38, 39). Our findings therefore complement the existing literature by demonstrating that there is no increase in serious myocardial or structural cardiovascular disease among predominantly ambulatory pregnant women.

### Mechanism

A potential explanation for the lack of increased severe complications may lie in the unique immunological milieu of pregnancy. Pregnancy induces a physiological state of immune modulation, characterized by a shift toward a Th2-type cytokine profile to promote fetal tolerance. This state often leads to the spontaneous remission of certain autoimmune diseases, such as rheumatoid arthritis (40, 41). It is possible that this dampened pro-inflammatory response serves as a protective mechanism against the “cytokine storm” typically associated with severe COVID-19-related myocardial injury. While non-pregnant patients may suffer from exuberant systemic inflammation leading to myocarditis, the immunomodulatory environment of pregnancy might mitigate this pathway, resulting in fewer objective cardiac events despite the presence of infection. Although the Th1/Th2 paradigm only partially explains the complex immunology of pregnancy, accumulating evidence indicates that successful pregnancy is accompanied by finely regulated innate and adaptive immune responses that may limit excessive inflammatory injury. Experimental and clinical studies of COVID-19 suggest that severe myocardial injury is largely driven by dysregulated immune activation, endothelial inflammation, and cytokine-mediated tissue damage. Therefore, the relatively controlled inflammatory environment of uncomplicated pregnancy may provide partial protection against clinically significant myocarditis in women with mild SARS-CoV-2 infection. Nevertheless, this hypothesis remains speculative and requires confirmation in prospective mechanistic studies (10, 18, 39, 41).

The study was underpowered to characterize the temporal distribution of rare cardiovascular events: with six events per group across the follow-up year, stratification by trimester and postpartum period yields no interpretable comparison (Supplementary Table 1 and Figure 2). In addition, the excess of recorded backache was accompanied by a baseline imbalance in the same symptom, so residual confounding in care-seeking or symptom-reporting behavior cannot be excluded.

### Clinical implications

Our findings are highly reassuring for clinicians, suggesting that in the absence of severe respiratory distress or “red-flag” clinical signs, COVID-19 infection does not necessitate a radical departure from standard obstetric-cardiac care. Although women with COVID-19 more frequently reported symptoms such as cough, backache, and, to a lesser extent, chest pain, these manifestations were not associated with progression to major adverse cardiovascular events.

### Strengths and limitations

A major strength of our study is the meticulous matching process, which included BMI category, demographic factors and socioeconomic status. This rigorous control for confounding factors reinforces our conclusion that COVID-19 in its ambulatory settings does not constitute a major cardiovascular risk factor during pregnancy. Additionally, the availability of comprehensive EHR data on all pregnant women including demographics, co-morbidities, and follow-up complications—provides a robust population-based perspective that is often missing from hospital-centric registries.

However, several limitations must be acknowledged. The retrospective nature of the analysis inherently carries risks of documentation bias. Because exposure was defined at the pregnancy level and follow-up was anchored to the estimated LMP, some recorded outcomes may have preceded the documented SARS-CoV-2 infection, precluding temporal or causal attribution of individual outcomes to infection. Detailed information regarding diagnostic investigations performed during each chest pain encounter (including electrocardiography, cardiac biomarkers, echocardiography, or adjudicated cardiac diagnoses) was not consistently available within the electronic health record database used for this study. Furthermore, while our community-based data is extensive, including inpatient diagnoses, it lacked detailed information on the in-hospital clinical course as dynamic hemodynamic, laboratory, imaging, and therapeutic changes occurring during hospitalization among the small subset of women requiring inpatient care. Nevertheless, longitudinal follow-up after hospitalization continued within LHS community services, where subsequent clinically recognized diagnoses and symptoms were documented in the EHR. Although this longitudinal ascertainment allows post-acute manifestations to be captured, the present study did not apply a formal Long-COVID definition and individual symptoms should not be interpreted as establishing this diagnosis. An additional limitation is that the study period encompassed several SARS-CoV-2 variant eras and evolving vaccination strategies. Because variant-specific viral data were unavailable, we were unable to determine whether cardiovascular outcomes differed according to infecting variant. Future studies should evaluate whether variant-specific pathogenicity influences maternal cardiovascular risk during pregnancy. Objective measures of disease severity, such as oxygen saturation or the need for respiratory support, were not recorded, so infection was classified by care setting rather than by measured severity. Because COVID-19 status was taken from the national registry rather than from hospital presentation, case ascertainment did not depend on whether a woman sought care, and the main residual source of misclassification is wholly asymptomatic infection among controls, which would bias the comparison toward the null. Eligibility required a documented second-trimester date, so pregnancies ending in first-trimester loss were not represented in either group, and the results apply to pregnancies reaching the second trimester. A comparison between the pre-Omicron and Omicron-dominant periods showed no material difference in cardiovascular outcomes (Supplementary Table 3).

## Conclusions

Our study found no statistically significant difference in the incidence of major cardiovascular complications between pregnant women with COVID-19 infection in ambulatory settings. However, the small number of observed cardiovascular events limits the ability to exclude a clinically meaningful increased risk. These findings support the use of standard clinical and cardiac monitoring protocols for pregnant women with predominantly ambulatory COVID-19.

## Data Availability

The data analyzed in this study is subject to the following licenses/restrictions: All the data is anonymized. Requests to access these datasets should be directed to idavarashvili@yahoo.com.

## References

[1] Chen R Zhang Y Huang L Cheng BH Xia ZY Meng QT. Safety and efficacy of different anesthetic regimens for parturients with COVID-19 undergoing cesarean delivery: a case series of 17 patients. Can J Anaesth. (2020) 67:655–63. 10.1007/s12630-020-01630-7 32180175 PMC7090434

[2] Chen H Guo J Wang C Luo F Yu X Zhang W. Clinical characteristics and intrauterine vertical transmission potential of COVID-19 infection in nine pregnant women: a retrospective review of medical records. Lancet. (2020) 395:809–15. 10.1016/S0140-6736(20)30360-3 32151335 PMC7159281

[3] Cucinotta D Vanelli M. WHO declares COVID-19 a pandemic. Acta Biomed. (2020) 91:157–60. 10.23750/abm.v91i1.9397 32191675 PMC7569573

[4] Mercedes BR Serwat A Naffaa L Ramirez N Khalid F Steward SB. New-onset myocardial injury in pregnant patients with coronavirus disease 2019: a case series of 15 patients. Am J Obstet Gynecol. (2021) 224:387.e1–e9. 10.1016/j.ajog.2020.10.031 33098814 PMC7577917

[5] Liu K Fang Y-Y Deng Y Liu W Wang M-F Ma J-P. Clinical characteristics of novel coronavirus cases in tertiary hospitals in Hubei province. Chin Med J (Engl). (2020) 133:1025–31. 10.1097/CM9.0000000000000744 32044814 PMC7147277

[6] Xu Z Shi L Wang Y Zhang J Huang L Zhang C. Pathological findings of COVID-19 associated with acute respiratory distress syndrome. Lancet Respir Med. (2020) 8:420–2. 10.1016/S2213-2600(20)30076-X 32085846 PMC7164771

[7] Liu Y Yang Y Zhang C Huang F Wang F Yuan J. Clinical and biochemical indexes from 2019-nCoV infected patients linked to viral loads and lung injury. Sci China Life Sci. (2020) 63:364–74. 10.1007/s11427-020-1643-8 32048163 PMC7088566

[8] Babapoor-Farrokhran S Gill D Walker J Rasekhi RT Bozorgnia B Amanullah A. Myocardial injury and COVID-19: possible mechanisms. Life Sci. (2020) 253:117723. 10.1016/j.lfs.2020.117723 32360126 PMC7194533

[9] Driggin E Madhavan MV Bikdeli B Chuich T Laracy J Biondi-Zoccai G. Cardiovascular considerations for patients, health care workers, and health systems during the COVID-19 pandemic. J Am Coll Cardiol. (2020) 75:2352–71. 10.1016/j.jacc.2020.03.031 32201335 PMC7198856

[10] Tajbakhsh A Gheibi Hayat SM Taghizadeh H Akbari A Inabadi M Savardashtaki A. COVID-19 and cardiac injury: clinical manifestations, biomarkers, mechanisms, diagnosis, treatment, and follow up. Expert Rev Anti Infect Ther. (2021) 19:345–57. 10.1080/14787210.2020.1822737 32921216

[11] Alhogbani T. Acute myocarditis associated with novel Middle East respiratory syndrome coronavirus. Ann Saudi Med. (2016) 36:78–80. 10.5144/0256-4947.2016.78 26922692 PMC6074274

[12] Welt FGP Shah PB Aronow HD Bortnick AE Henry TD Sherwood MW. Catheterization laboratory considerations during the coronavirus (COVID-19) pandemic: from the ACC’s interventional council and the society for cardiovascular angiography and interventions. J Am Coll Cardiol. (2020) 75:2372–5. 10.1016/j.jacc.2020.03.021 32199938 PMC7270593

[13] Shi S Qin M Shen B Cai Y Liu T Yang F. Association of cardiac injury with mortality in hospitalized patients with COVID-19 in Wuhan, China. JAMA Cardiol. (2020) 5:802–10. 10.1001/jamacardio.2020.0950 32211816 PMC7097841

[14] Guo T Fan Y Chen M Wu X Zhang L He T. Cardiovascular implications of fatal outcomes of patients with coronavirus disease 2019 (COVID-19). JAMA Cardiol. (2020) 5:811–8. 10.1001/jamacardio.2020.1017 32219356 PMC7101506

[15] Buzon J Roignot O Lemoine S Perez-Martin A Buffet P Rioufol G. Takotsubo cardiomyopathy triggered by influenza A virus. Intern Med. (2015) 54:2017–9. 10.2169/internalmedicine.54.3606 26278294

[16] Corrales-Medina VF Alvarez KN Weissfeld LA Angus DC Chirinos JA Chang C-CH. Association between hospitalization for pneumonia and subsequent risk of cardiovascular disease. JAMA. (2015) 313:264–74. 10.1001/jama.2014.18229 25602997 PMC4687729

[17] Xie Y Xu E Bowe B Al-Aly Z. Long-term cardiovascular outcomes of COVID-19. Nat Med. (2022) 28:583–90. 10.1038/s41591-022-01689-3 35132265 PMC8938267

[18] Bonaventura A Vecchié A Dagna L Martinod K Dixon DL Van Tassell BW. Endothelial dysfunction and immunothrombosis as key pathogenic mechanisms in COVID-19. Nat Rev Immunol. (2021) 21:319–29. 10.1038/s41577-021-00536-9 33824483 PMC8023349

[19] Chen C Zhou Y Wang DW. SARS-CoV-2: a potential novel etiology of fulminant myocarditis. Herz. (2020) 45:230–2. 10.1007/s00059-020-04909-z 32140732 PMC7080076

[20] Estensen ME Beitnes JO Grindheim G Aaberge L Smiseth OA Henriksen T. Altered maternal left ventricular contractility and function during normal pregnancy. Ultrasound Obstet Gynecol. (2013) 41:659–66. 10.1002/uog.12296 23001841

[21] Ouzounian JG Elkayam U. Physiologic changes during normal pregnancy and delivery. Cardiol Clin. (2012) 30:317–29. 10.1016/j.ccl.2012.05.004 22813360

[22] Kamel H Roman MJ Pitcher A Devereux RB. Pregnancy and the risk of aortic dissection or rupture: a cohort-crossover analysis. Circulation. (2016) 134:527–33. 10.1161/CIRCULATIONAHA.116.021594 27492904 PMC4987245

[23] Graves CR Davis SF. Cardiovascular complications in pregnancy: it is time for action. Circulation. (2018) 137:1213–5. 10.1161/CIRCULATIONAHA.117.031592 29459362

[24] Ellington S Strid P Tong VT Woodworth K Galang RR Zambrano LD. Characteristics of women of reproductive age with laboratory-confirmed SARS-CoV-2 infection by pregnancy status—united States, January 22–June 7, 2020. MMWR Morb Mortal Wkly Rep. (2020) 69:769–75. 10.15585/mmwr.mm6925a1 32584795 PMC7316319

[25] Deng J Ma Y Liu Q Du M Liu M Liu J. Association of infection with different SARS-CoV-2 variants during pregnancy with maternal and perinatal outcomes: a systematic review and meta-analysis. Int J Environ Res Public Health. (2022) 19:15932. 10.3390/ijerph192315932 36498007 PMC9740636

[26] Long B Brady WJ Koyfman A Gottlieb M. Cardiovascular complications in COVID-19. Am J Emerg Med. (2020) 38:1504–7. 10.1016/j.ajem.2020.04.048 32317203 PMC7165109

[27] Zahid S Agrawal A Rai D Khan MZ Michos ED. Cardiovascular complications associated with COVID-19 during delivery hospitalizations in pandemic year 2020. JACC Adv. (2023) 2:100386. 10.1016/j.jacadv.2023.100386 38938230 PMC11198635

[28] Metz TD Clifton RG Hughes BL Sandoval GJ Grobman WA Saade GR. Association of SARS-CoV-2 infection with serious maternal morbidity and mortality from obstetric complications. JAMA. (2022) 327:748–59. 10.1001/jama.2022.1190 35129581 PMC8822445

[29] Ferrara A Hedderson MM Zhu Y Avalos LA Kuzniewicz MW Myers LC. Perinatal complications in individuals in California with or without SARS-CoV-2 infection during pregnancy. JAMA Intern Med. (2022) 182:503–12. 10.1001/jamainternmed.2022.0330 35311909 PMC8938896

[30] Bikdeli B Krishnathasan D Khairani CD Bejjani A Davies J Porio N. Low absolute risk of thrombotic and cardiovascular events in outpatient pregnant women with COVID-19. Thromb Res. (2024) 237:209–15. 10.1016/j.thromres.2024.02.021 38677791

[31] Chen T Wu D Chen H Yan W Yang D Chen G. Clinical characteristics of 113 deceased patients with coronavirus disease 2019: retrospective study. Br Med J. (2020) 368:m1091. 10.1136/bmj.m1091 PMC7190011 32217556

[32] Huang C Wang Y Li X Ren L Zhao J Hu Y. Clinical features of patients infected with 2019 novel coronavirus in Wuhan, China. Lancet. (2020) 395:497–506. 10.1016/S0140-6736(20)30183-5 PMC7159299 31986264

[33] Zhou F Yu T Du R Fan G Liu Y Liu Z. Clinical course and risk factors for mortality of adult inpatients with COVID-19 in Wuhan, China: a retrospective cohort study. Lancet. (2020) 395:1054–62. 10.1016/S0140-6736(20)30566-3 32171076 PMC7270627

[34] Wang D Hu B Hu C Zhu F Liu X Zhang J. Clinical characteristics of 138 hospitalized patients with 2019 novel coronavirus-infected pneumonia in Wuhan, China. JAMA. (2020) 323:1061–9. 10.1001/jama.2020.1585 32031570 PMC7042881

[35] Wu Z McGoogan JM. Characteristics of and important lessons from the coronavirus disease 2019 (COVID-19) outbreak in China: summary of a report of 72,314 cases from the Chinese center for disease control and prevention. JAMA. (2020) 323:1239–42. 10.1001/jama.2020.2648 32091533

[36] Ruan Q Yang K Wang W Jiang L Song J. Clinical predictors of mortality due to COVID-19 based on an analysis of data of 150 patients from Wuhan, China. Intensive Care Med. (2020) 46:846–8. 10.1007/s00134-020-05991-x 32125452 PMC7080116

[37] Raman B Bluemke DA Lüscher TF Neubauer S. Long COVID: post-acute sequelae of COVID-19 with a cardiovascular focus. Eur Heart J. (2022) 43:1157–72. 10.1093/eurheartj/ehac031 35176758 PMC8903393

[38] Bruno AM Horns JJ Metz TD. Coronavirus disease 2019 (COVID-19) and venous thromboembolism during pregnancy and postpartum. Obstet Gynecol. (2024) 143(1):139–42. 10.1097/AOG.0000000000005387 38096554 PMC10730116

[39] Örtqvist AK Söderling J Magnus MC Urhoj SK Håberg SE Stephansson O. COVID-19 infection increases the risk of venous thromboembolism during pregnancy and the postpartum period. Eur J Epidemiol. (2025) 40:779–88. 10.1007/s10654-025-01268-z PMC12304068 40643819

[40] Nelson JL Østensen M. Pregnancy and rheumatoid arthritis. Rheum Dis Clin North Am. (1997) 23:195–212. 10.1016/S0889-857X(05)70323-9 9031383

[41] Østensen M Villiger PM. The remission of rheumatoid arthritis during pregnancy. Semin Immunopathol. (2007) 29:185–91. 10.1007/s00281-007-0072-5 17621703

