# Supplementary material for: Cardiovascular complications as a consequence of coronavirus infection in pregnant women managed in ambulatory settings
Source: Front Cardiovasc Med. 2026 Oct 15;13:1907396. doi: 10.3389/fcvm.2026.1907396 (PMC13716656; doi:10.3389/fcvm.2026.1907396)
Supplement: Supplementary file 1 [file Datasheet1.pdf]

Supplementary Table S1.

Cardiovascular and symptom outcomes among COVID-19-exposed pregnancies and matched controls, stratified by gestational and postpartum period. Each outcome was defined as ≥1 recorded clinical encounter for the condition within the stated interval, between 30 days after the estimated last menstrual period (LMP) and 365 days thereafter. Counts are numbers of women (percentage of the 4,068 women in each group); incidence rates are per 1,000 person-years (py) with exact Poisson 95% confidence intervals; odds ratios (OR) compare the exposed with the control group by period, with exact (conditional) 95% confidence intervals and two-sided Fisher exact p-values.

Outcome / period	COVID-19 n (%)	Controls n (%)	COVID-19 rate/1000py (95% CI)	Controls rate/1000py (95% CI)	OR (95% CI)	p
**MACE (composite)**						
First trimester	5 (0.12%)	2 (0.05%)	6.6 (2.14 to 15.41)	2.6 (0.32 to 9.54)	2.50 (0.41 to 26.28)	0.453
Second trimester	4 (0.10%)	1 (0.02%)	3.7 (1.00 to 9.39)	0.9 (0.02 to 5.11)	4.00 (0.40 to 197.18)	0.375
Third trimester	0 (0.00%)	2 (0.05%)	0.0 (0.00 to 4.14)	2.2 (0.27 to 8.12)	0.00 (0.00 to 5.33)	0.500
Early postpartum	2 (0.05%)	2 (0.05%)	4.3 (0.52 to 15.44)	4.3 (0.52 to 15.44)	1.00 (0.07 to 13.80)	1.000
Late postpartum	1 (0.02%)	3 (0.07%)	1.9 (0.05 to 10.62)	5.7 (1.18 to 16.68)	0.33 (0.01 to 4.15)	0.625
**Whole window**	6 (0.15%)	6 (0.15%)	1.6 (0.59 to 3.50)	1.6 (0.59 to 3.50)	1.00 (0.27 to 3.74)	1.000
**Myocarditis**						
First trimester	0 (0.00%)	1 (0.02%)	0.0 (0.00 to 4.87)	1.3 (0.03 to 7.36)	0.00 (0.00 to 39.00)	1.000
Second trimester	0 (0.00%)	1 (0.02%)	0.0 (0.00 to 3.38)	0.9 (0.02 to 5.11)	0.00 (0.00 to 39.00)	1.000
Third trimester	0 (0.00%)	0 (0.00%)	0.0 (0.00 to 4.14)	0.0 (0.00 to 4.15)	–	1.000
Early postpartum	0 (0.00%)	0 (0.00%)	0.0 (0.00 to 7.89)	0.0 (0.00 to 7.89)	–	1.000
Late postpartum	0 (0.00%)	0 (0.00%)	0.0 (0.00 to 7.03)	0.0 (0.00 to 7.02)	–	1.000
**Whole window**	0 (0.00%)	2 (0.05%)	0.0 (0.00 to 0.99)	0.5 (0.06 to 1.94)	0.00 (0.00 to 5.32)	0.500
**Cardiomyopathy**						
First trimester	1 (0.02%)	0 (0.00%)	1.3 (0.03 to 7.36)	0.0 (0.00 to 4.87)	–	1.000
Second trimester	1 (0.02%)	0 (0.00%)	0.9 (0.02 to 5.11)	0.0 (0.00 to 3.38)	–	1.000
Third trimester	0 (0.00%)	0 (0.00%)	0.0 (0.00 to 4.14)	0.0 (0.00 to 4.15)	–	1.000
Early postpartum	0 (0.00%)	1 (0.02%)	0.0 (0.00 to 7.89)	2.1 (0.05 to 11.91)	0.00 (0.00 to 39.00)	1.000
Late postpartum	0 (0.00%)	1 (0.02%)	0.0 (0.00 to 7.03)	1.9 (0.05 to 10.60)	0.00 (0.00 to 39.00)	1.000
**Whole window**	1 (0.02%)	1 (0.02%)	0.3 (0.01 to 1.49)	0.3 (0.01 to 1.49)	1.00 (0.01 to 78.52)	1.000
**Atrial fibrillation**						
First trimester	0 (0.00%)	0 (0.00%)	0.0 (0.00 to 4.87)	0.0 (0.00 to 4.87)	–	1.000
Second trimester	0 (0.00%)	0 (0.00%)	0.0 (0.00 to 3.38)	0.0 (0.00 to 3.38)	–	1.000
Third trimester	0 (0.00%)	0 (0.00%)	0.0 (0.00 to 4.14)	0.0 (0.00 to 4.15)	–	1.000
Early postpartum	0 (0.00%)	0 (0.00%)	0.0 (0.00 to 7.89)	0.0 (0.00 to 7.89)	–	1.000
Late postpartum	0 (0.00%)	0 (0.00%)	0.0 (0.00 to 7.03)	0.0 (0.00 to 7.02)	–	1.000
**Whole window**	0 (0.00%)	0 (0.00%)	0.0 (0.00 to 0.99)	0.0 (0.00 to 0.99)	–	1.000
**Atrial flutter**						
First trimester	0 (0.00%)	0 (0.00%)	0.0 (0.00 to 4.87)	0.0 (0.00 to 4.87)	–	1.000
Second trimester	0 (0.00%)	0 (0.00%)	0.0 (0.00 to 3.38)	0.0 (0.00 to 3.38)	–	1.000

Outcome / period	COVID-19 n (%)	Controls n (%)	COVID-19 rate/1000py (95% CI)	Controls rate/1000py (95% CI)	OR (95% CI)	p
Third trimester	0 (0.00%)	0 (0.00%)	0.0 (0.00 to 4.14)	0.0 (0.00 to 4.15)	–	1.000
Early postpartum	0 (0.00%)	0 (0.00%)	0.0 (0.00 to 7.89)	0.0 (0.00 to 7.89)	–	1.000
Late postpartum	0 (0.00%)	0 (0.00%)	0.0 (0.00 to 7.03)	0.0 (0.00 to 7.02)	–	1.000
**Whole window**	0 (0.00%)	0 (0.00%)	0.0 (0.00 to 0.99)	0.0 (0.00 to 0.99)	–	1.000
**Congestive heart failure**						
First trimester	0 (0.00%)	0 (0.00%)	0.0 (0.00 to 4.87)	0.0 (0.00 to 4.87)	–	1.000
Second trimester	0 (0.00%)	0 (0.00%)	0.0 (0.00 to 3.38)	0.0 (0.00 to 3.38)	–	1.000
Third trimester	0 (0.00%)	0 (0.00%)	0.0 (0.00 to 4.14)	0.0 (0.00 to 4.15)	–	1.000
Early postpartum	0 (0.00%)	1 (0.02%)	0.0 (0.00 to 7.89)	2.1 (0.05 to 11.91)	0.00 (0.00 to 39.00)	1.000
Late postpartum	0 (0.00%)	1 (0.02%)	0.0 (0.00 to 7.03)	1.9 (0.05 to 10.60)	0.00 (0.00 to 39.00)	1.000
**Whole window**	0 (0.00%)	1 (0.02%)	0.0 (0.00 to 0.99)	0.3 (0.01 to 1.49)	0.00 (0.00 to 39.00)	1.000
**Pulmonary embolism**						
First trimester	1 (0.02%)	0 (0.00%)	1.3 (0.03 to 7.36)	0.0 (0.00 to 4.87)	–	1.000
Second trimester	1 (0.02%)	0 (0.00%)	0.9 (0.02 to 5.11)	0.0 (0.00 to 3.38)	–	1.000
Third trimester	0 (0.00%)	0 (0.00%)	0.0 (0.00 to 4.14)	0.0 (0.00 to 4.15)	–	1.000
Early postpartum	0 (0.00%)	0 (0.00%)	0.0 (0.00 to 7.89)	0.0 (0.00 to 7.89)	–	1.000
Late postpartum	0 (0.00%)	0 (0.00%)	0.0 (0.00 to 7.03)	0.0 (0.00 to 7.02)	–	1.000
**Whole window**	1 (0.02%)	0 (0.00%)	0.3 (0.01 to 1.49)	0.0 (0.00 to 0.99)	–	1.000
**Deep venous thrombosis**						
First trimester	3 (0.07%)	0 (0.00%)	4.0 (0.82 to 11.58)	0.0 (0.00 to 4.87)	–	0.250
Second trimester	2 (0.05%)	0 (0.00%)	1.8 (0.22 to 6.62)	0.0 (0.00 to 3.38)	–	0.500
Third trimester	0 (0.00%)	1 (0.02%)	0.0 (0.00 to 4.14)	1.1 (0.03 to 6.26)	0.00 (0.00 to 39.01)	1.000
Early postpartum	2 (0.05%)	0 (0.00%)	4.3 (0.52 to 15.44)	0.0 (0.00 to 7.89)	–	0.500
Late postpartum	1 (0.02%)	1 (0.02%)	1.9 (0.05 to 10.62)	1.9 (0.05 to 10.60)	1.00 (0.01 to 78.52)	1.000
**Whole window**	4 (0.10%)	1 (0.02%)	1.1 (0.29 to 2.74)	0.3 (0.01 to 1.49)	4.00 (0.40 to 197.18)	0.375
**Chest pain**						
First trimester	19 (0.47%)	16 (0.39%)	25.1 (15.10 to 39.18)	21.1 (12.08 to 34.31)	1.19 (0.58 to 2.47)	0.735
Second trimester	26 (0.64%)	30 (0.74%)	23.8 (15.56 to 34.91)	27.5 (18.55 to 39.25)	0.87 (0.49 to 1.52)	0.688
Third trimester	25 (0.62%)	12 (0.30%)	28.1 (18.17 to 41.45)	13.5 (6.97 to 23.57)	2.09 (1.01 to 4.57)	0.033
Early postpartum	8 (0.20%)	5 (0.12%)	17.1 (7.38 to 33.70)	10.7 (3.47 to 24.94)	1.60 (0.46 to 6.23)	0.581
Late postpartum	13 (0.32%)	7 (0.17%)	24.8 (13.20 to 42.39)	13.3 (5.36 to 27.45)	1.86 (0.69 to 5.51)	0.263
**Whole window**	86 (2.11%)	65 (1.60%)	23.1 (18.44 to 28.47)	17.4 (13.45 to 22.20)	1.33 (0.95 to 1.87)	0.100
**Backache**						
First trimester	90 (2.21%)	100 (2.46%)	118.8 (95.56 to 146.07)	132.0 (107.43 to 160.59)	0.90 (0.67 to 1.21)	0.509
Second trimester	238 (5.85%)	204 (5.01%)	218.1 (191.28 to 247.65)	187.0 (162.20 to 214.48)	1.18 (0.97 to 1.43)	0.106
Third trimester	226 (5.57%)	193 (4.76%)	253.8 (221.81 to 289.17)	217.0 (187.45 to 249.85)	1.18 (0.97 to 1.45)	0.098
Early postpartum	57 (1.40%)	49 (1.20%)	121.8 (92.29 to 157.87)	104.8 (77.49 to 138.49)	1.17 (0.78 to 1.75)	0.494

Outcome / period	COVID-19 n (%)	Controls n (%)	COVID-19 rate/1000py (95% CI)	Controls rate/1000py (95% CI)	OR (95% CI)	p
Late postpartum	73 (1.79%)	72 (1.77%)	139.2 (109.11 to 175.03)	137.0 (107.21 to 172.55)	1.01 (0.72 to 1.43)	1.000
**Whole window**	562 (13.82%)	480 (11.80%)	150.6 (138.43 to 163.61)	128.7 (117.40 to 140.69)	1.20 (1.05 to 1.37)	0.007
**Headache**						
First trimester	69 (1.70%)	64 (1.57%)	91.1 (70.89 to 115.30)	84.5 (65.08 to 107.91)	1.08 (0.75 to 1.55)	0.727
Second trimester	100 (2.46%)	92 (2.26%)	91.6 (74.56 to 111.46)	84.3 (67.98 to 103.42)	1.09 (0.81 to 1.47)	0.609
Third trimester	56 (1.38%)	45 (1.11%)	62.9 (47.51 to 81.68)	50.6 (36.90 to 67.70)	1.25 (0.83 to 1.90)	0.273
Early postpartum	29 (0.71%)	22 (0.54%)	62.0 (41.52 to 89.04)	47.0 (29.47 to 71.21)	1.32 (0.73 to 2.42)	0.400
Late postpartum	25 (0.61%)	21 (0.52%)	47.7 (30.85 to 70.37)	40.0 (24.74 to 61.09)	1.19 (0.64 to 2.24)	0.658
**Whole window**	249 (6.12%)	210 (5.16%)	66.7 (58.70 to 75.56)	56.3 (48.93 to 64.43)	1.20 (0.99 to 1.45)	0.068
**Cough**						
First trimester	67 (1.65%)	37 (0.91%)	88.5 (68.56 to 112.35)	48.9 (34.40 to 67.34)	1.82 (1.20 to 2.81)	0.004
Second trimester	118 (2.90%)	79 (1.94%)	108.1 (89.51 to 129.50)	72.4 (57.33 to 90.24)	1.51 (1.12 to 2.04)	0.006
Third trimester	93 (2.29%)	61 (1.50%)	104.5 (84.31 to 127.96)	68.6 (52.46 to 88.10)	1.54 (1.10 to 2.17)	0.009
Early postpartum	24 (0.59%)	16 (0.39%)	51.3 (32.87 to 76.34)	34.2 (19.55 to 55.55)	1.50 (0.76 to 3.03)	0.267
Late postpartum	15 (0.37%)	23 (0.57%)	28.6 (16.01 to 47.18)	43.8 (27.75 to 65.67)	0.65 (0.32 to 1.30)	0.255
**Whole window**	280 (6.88%)	198 (4.87%)	75.0 (66.51 to 84.37)	53.1 (45.93 to 61.00)	1.45 (1.19 to 1.75)	<0.001

MACE, major adverse cardiovascular events (composite of myocarditis, cardiomyopathy, atrial fibrillation or flutter, myocardial infarction, stroke or transient ischaemic attack, congestive heart failure, pulmonary embolism, and deep venous thrombosis). Third-trimester intervals contain 11 women whose pregnancy ended before 28w0d and who therefore contributed no third-trimester person-time. A dash denotes an undefined estimate (no events in either group).
