# Supplementary material for: Cardiovascular complications as a consequence of coronavirus infection in pregnant women managed in ambulatory settings
Source: Front Cardiovasc Med. 2026 Oct 15;13:1907396. doi: 10.3389/fcvm.2026.1907396 (PMC13716656; doi:10.3389/fcvm.2026.1907396)
Supplement: Supplementary file 2 [file Datasheet2.pdf]

Supplementary Table S2.

Decomposition of outcome ascertainment. For each outcome the table separates the lifetime prevalence recorded before the exposure window (any encounter before LMP + 30 days) from events occurring within the follow-up window, and from first-ever occurrences within the window. This distinguishes pre-existing between-group differences from those arising during or after pregnancy, and shows why cumulative-prevalence ascertainment (used in the original submission) can differ from within-window ascertainment. Values are percentages of the 4,068 women in each group; p-values are two-sided Fisher exact tests.

Outcome	COVID-19 ever before LMP+30d	Controls ever before LMP+30d	p (before)	COVID-19 any in window	Controls any in window	p (in window)	COVID-19 first-ever in window	Controls first-ever in window	p (incident)
MACE (composite)	0.22%	0.22%	1.000	0.15%	0.15%	1.000	0.05%	0.07%	1.000
Myocarditis	0.02%	0.07%	0.625	0.00%	0.05%	0.500	0.00%	0.00%	1.000
Cardiomyopathy	0.02%	0.00%	1.000	0.02%	0.02%	1.000	0.00%	0.02%	1.000
Atrial fibrillation	0.02%	0.00%	1.000	0.00%	0.00%	1.000	0.00%	0.00%	1.000
Atrial flutter	0.00%	0.02%	1.000	0.00%	0.00%	1.000	0.00%	0.00%	1.000
Congestive heart failure	0.00%	0.00%	1.000	0.00%	0.02%	1.000	0.00%	0.02%	1.000
Pulmonary embolism	0.05%	0.02%	1.000	0.02%	0.00%	1.000	0.00%	0.00%	1.000
Deep venous thrombosis	0.10%	0.07%	1.000	0.10%	0.02%	0.375	0.05%	0.02%	1.000
Chest pain	17.65%	16.45%	0.157	2.14%	1.60%	0.085	1.38%	0.93%	0.077
Backache	49.46%	46.12%	0.003	13.86%	11.85%	0.007	3.98%	3.49%	0.267
Headache	48.55%	46.78%	0.115	6.15%	5.16%	0.061	2.04%	1.60%	0.158
Cough	37.61%	38.45%	0.451	6.93%	4.87%	<0.001	3.00%	2.21%	0.031

The chest pain difference reported in the original submission reflected the “ever before LMP+30d” and cumulative columns, which were already higher in the exposed group before conception (17.65% vs 16.45%) rather than the within-window or incident columns. Cough, by contrast, was balanced at baseline and elevated within the window, indicating ascertainment sensitive to infection-attributable morbidity.
