# Supplementary material for: Cardiovascular complications as a consequence of coronavirus infection in pregnant women managed in ambulatory settings
Source: Front Cardiovasc Med. 2026 Oct 15;13:1907396. doi: 10.3389/fcvm.2026.1907396 (PMC13716656; doi:10.3389/fcvm.2026.1907396)
Supplement: Supplementary file 3 [file Datasheet3.pdf]

Supplementary Table S3.

Sensitivity analyses: whole-window outcomes in the full matched cohort and in prespecified subgroups. Subgroups comprise matched pairs in which both women were unvaccinated, and pairs stratified by pandemic period (pre-Omicron, estimated LMP before 1 January 2022; Omicron-dominant, from that date). Because number of pre-pregnancy vaccine doses and LMP quarter were exact matching variables, each stratum is internally homogeneous for both, so subsetting preserves the 1:1 matching. Counts are numbers of women (percentage of the group); odds ratios (OR) compare exposed with control with exact (conditional) 95% confidence intervals and two-sided Fisher exact p-values. Because vaccination was withheld from pregnant women until mid-2021, the unvaccinated and pre-Omicron strata overlap substantially and are not independent.

Outcome / subset	Matched pairs	COVID-19 n (%)	Controls n (%)	OR (95% CI)	p
**MACE (composite)**					
**Full cohort**	4,068	6 (0.15%)	6 (0.15%)	1.00 (0.27 to 3.74)	1
Unvaccinated	2,268	3 (0.13%)	5 (0.22%)	0.60 (0.09 to 3.09)	0.726
Pre-Omicron (LMP<2022)	3,176	5 (0.16%)	5 (0.16%)	1.00 (0.23 to 4.35)	1
Omicron (LMP>=2022)	892	1 (0.11%)	1 (0.11%)	1.00 (0.01 to 78.58)	1
**Chest pain**					
**Full cohort**	4,068	87 (2.14%)	65 (1.60%)	1.35 (0.96 to 1.89)	0.085
Unvaccinated	2,268	54 (2.38%)	39 (1.72%)	1.39 (0.90 to 2.17)	0.142
Pre-Omicron (LMP<2022)	3,176	66 (2.08%)	52 (1.64%)	1.27 (0.87 to 1.88)	0.227
Omicron (LMP>=2022)	892	21 (2.35%)	13 (1.46%)	1.63 (0.77 to 3.57)	0.225
**Cough**					
**Full cohort**	4,068	282 (6.93%)	198 (4.87%)	1.46 (1.20 to 1.76)	<0.001
Unvaccinated	2,268	147 (6.48%)	87 (3.84%)	1.74 (1.31 to 2.31)	<0.001
Pre-Omicron (LMP<2022)	3,176	214 (6.74%)	145 (4.57%)	1.51 (1.21 to 1.89)	<0.001
Omicron (LMP>=2022)	892	68 (7.62%)	53 (5.94%)	1.31 (0.89 to 1.93)	0.187
**Backache**					
**Full cohort**	4,068	564 (13.86%)	482 (11.85%)	1.20 (1.05 to 1.37)	0.007
Unvaccinated	2,268	268 (11.82%)	221 (9.74%)	1.24 (1.02 to 1.51)	0.028
Pre-Omicron (LMP<2022)	3,176	413 (13.00%)	339 (10.67%)	1.25 (1.07 to 1.46)	0.005
Omicron (LMP>=2022)	892	151 (16.93%)	143 (16.03%)	1.07 (0.82 to 1.38)	0.655
**Headache**					
**Full cohort**	4,068	250 (6.15%)	210 (5.16%)	1.20 (0.99 to 1.46)	0.061
Unvaccinated	2,268	136 (6.00%)	96 (4.23%)	1.44 (1.10 to 1.91)	0.008
Pre-Omicron (LMP<2022)	3,176	192 (6.05%)	157 (4.94%)	1.24 (0.99 to 1.55)	0.061
Omicron (LMP>=2022)	892	58 (6.50%)	53 (5.94%)	1.10 (0.74 to 1.65)	0.695

The composite cardiovascular estimate remained close to unity in every subset, with confidence intervals widening as the number of events fell; no subgroup revealed an excess obscured in the pooled analysis. Cough remained elevated across subsets. Cardiovascular event counts within subgroups were too small for precise subgroup-specific estimation; these analyses are reported to show stability of the primary result.
